# Hepatitis B virus infection among the tribal and particularly vulnerable tribal population from an eastern state of India: Findings from the serosurvey in seven tribal dominated districts, 2021–2022

**DOI:** 10.3389/fmicb.2023.1039696

**Published:** 2023-03-06

**Authors:** Haimanti Bhattacharya, Debaprasad Parai, Subrat Kumar Sahoo, Aparjita Swain, Matrujyoti Pattnaik, Ira Mohapatra, Hariram Choudhary, Girish Chandra Dash, Nausaba Akhtar, Jaya Singh Kshatri, Debdutta Bhattacharya, Sanghamitra Pati

**Affiliations:** Department of Microbiology, ICMR – Regional Medical Research Centre, Bhubaneswar, Odisha, India

**Keywords:** HBV, PVTGs, tribal population, serosurvey, viral load

## Abstract

The Global Health Sector Strategy on viral hepatitis (2016–2021) endorsed by the World Health Assembly in 2016, called for the elimination of viral hepatitis as a public health threat by 2030. Odisha, an eastern state of India, has the third-highest percentage of tribal population in the country and limited information is available regarding the prevalence of HBsAg among them. The present study was undertaken to estimate the seroprevalence of hepatitis B surface antigen as well as HBV DNA almost after 12 years of the first prevalence study of HBsAg among the tribal community of Odisha. The present study attempted to estimate the prevalence of HBsAg among the 35 Scheduled tribal (ST) communities and 5 Particularly Vulnerable Tribal Group (PVTG) using the 2,737 number of sera collected as part of a statewide COVID-19 serosurvey, among the tribal populations of Odisha (residing in 7 districts) aged 6–75 years. HBsAg positivity ranged between 1.79 and 2.94% across various age groups. 42.9% of HBsAg positive individuals showed the presence of HBV DNA and the high viral load was 0.10 × 10^2^–6.84 × 10^8^ IU/mL, indicating a high potential to transmit the virus. The HBsAg positivity was 14.18 and 6.06% among the PVTGs, Kutia Khond and Paudi Bhuyan, who were first time surveyed for HBsAg prevalence. The present study documents the prevalence of HBsAg among the major tribal population residing in the eastern state of the country and highlights the need for a statewide survey of Hepatitis B infection and risk factors, coverage and impact of the Hepatitis B vaccination program introduced in 2010–2011 in Odisha among the ST and PVTG population of the state.

## 1. Introduction

With a prevalence of 3–4.2% of Hepatitis B surface antigen (HBsAg) and 40 million HBV carriers, India ranks in the intermediate endemic zone for the Hepatitis B virus (HBV) infection in the world (WHO Factsheet-b- World Hepatitis Day, 2016). The Global Health Sector Strategy on viral hepatitis (2016–2021) endorsed by the World Health Assembly in 2016, called for the elimination of viral hepatitis as a public health threat by 2030 (World Health Organization, 2016). WHO recommends HB vaccination at birth followed by two or three doses to tackle the burden of hepatitis B (World Health Organization, 2019). A marked reduction in the prevalence of chronic HB virus infection, as well as hepatocellular cancer, has been observed in several countries after the introduction of HB vaccination (Chang et al., 1997; Liang et al., 2009).

Hepatitis B surface antigen, HBsAg (“Australian antigen” component), characteristically appears in the serum of the majority of patients with acute hepatitis B during the first few days of the illness and is the most important serum marker for diagnosing HBV infection. The presence of HBsAg indicates infection and the person is infected as long as HBsAg or HBV DNA can be detected in the blood (Bamdad and Yari, 2022). Odisha, a state in the eastern region of India, is a home to 62 different tribal community and 13 Particularly Vulnerable Tribal Group (PVTG). A Particularly vulnerable tribal group or PVTG previously known as a Primitive tribal group is a sub-classification of Scheduled Tribe or section of a Scheduled Tribe that is considered more vulnerable than a regular Scheduled Tribe. The PVTG list was created by the Indian Government with the purpose of better improving the living standards of endangered tribal groups based on priority. The state has the third-highest percentage of tribal population in the country (as per census 2011) which constitutes 22.85% of the total population of the state and 9.17% of the total tribal population of the country (ST & SC Development, Minorities & Backward Classes Welfare Department, 2022). Although the tribal population of Odisha accounts for a major proportion of the total population of the state, limited information is available regarding the prevalence of HBsAg among them. Previous studies documented a high prevalence of HBsAg (ranging from 2 to 65%) among the tribal population which also includes the PVTGs residing in different states like Andaman and Nicobar Islands, Arunachal Pradesh, and Jharkhand (Biswas et al., 2007; Bhattacharya et al., 2015; Sharma et al., 2019). HBV infection is one of the leading causes of death among the population worldwide as chronic HBV infection results in severe liver damage that leads to hepatitis, fibrosis, liver cirrhosis, and hepatocellular carcinoma (Dwibedi et al., 2014; Li et al., 2019). In Odisha, except for a study conducted a decade back, there are no published data on the prevalence of Hepatitis B infection among the tribal community of the state. The study undertaken between the year 2006 and 2010 revealed the prevalence of HBsAg among five PTGs (Lodha, Saora, Khadia, Mankidia, and Juanga) as 0.8, 0.9, 0.9, 3.7, and 1.7%, respectively, (Dwibedi et al., 2014). Conducting state-level surveys, however, is resource-intensive. We conducted a, among the tribal populations of Odisha (residing in 7 districts) aged 6–75 years to estimate the prevalence of antibody against virus. The sera collected from tribal population collected as part of the state wide representative COVID-19 serosurvey during September 2021 (Kshatri et al., 2022) were subsequently tested to estimate the seroprevalence of hepatitis B surface antigen as well as HBV DNA almost after 12 years of the first prevalence study of HBsAg among the tribal community of Odisha.

## 2. Methodology

### 2.1. Study setting and study design

**Figure 1:**
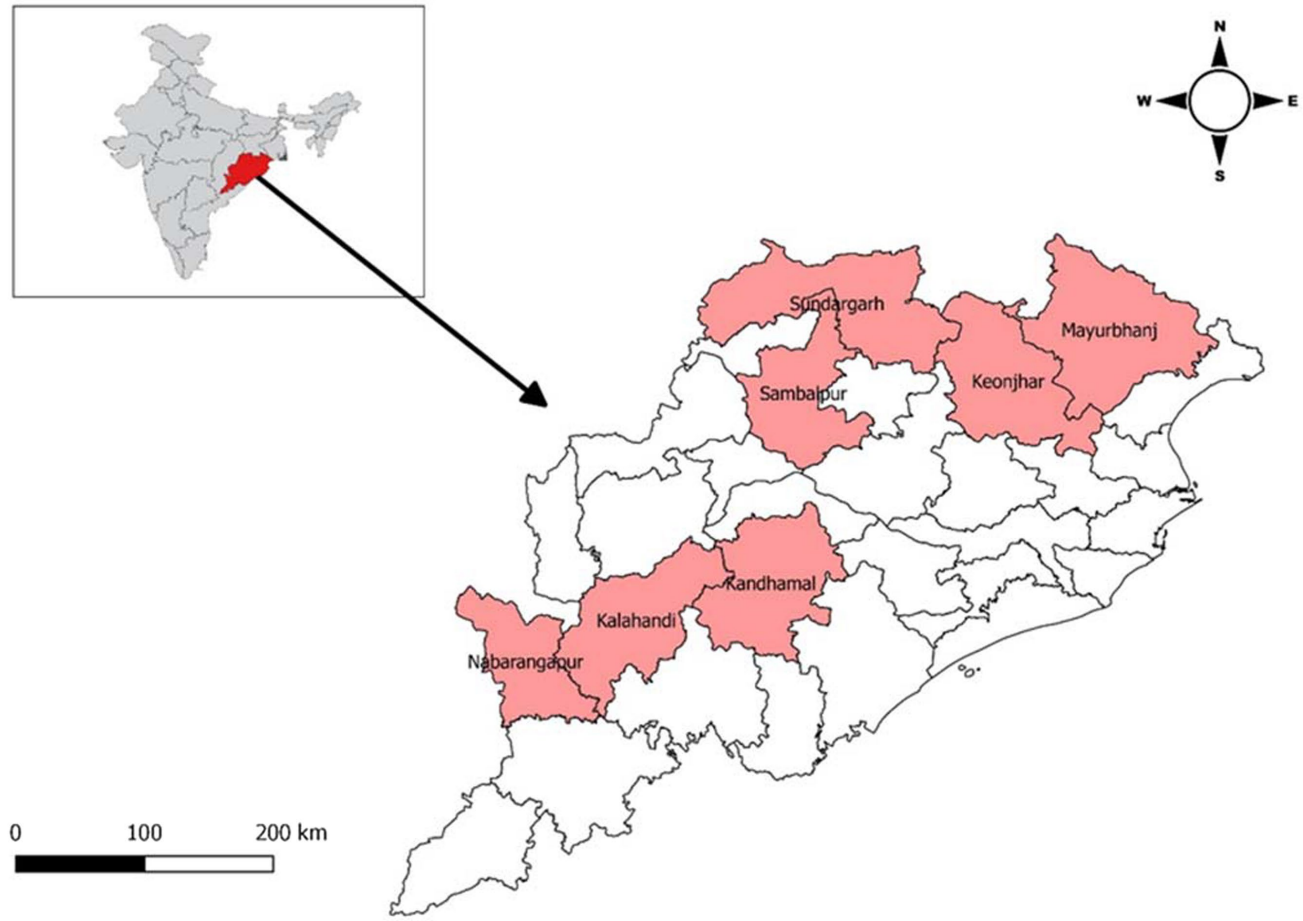
Heat map of the districts included in the study.

Odisha is a state which lies in the eastern part of India. Odisha has one of the largest Scheduled Tribes population (22.85% of ST population) with 62 Scheduled Tribes and 13 Particularly Vulnerable Tribal Groups (PVTGs). A majority of these tribal population live isolated in forest and hilly areas and are generally considered socially and economically marginalized. These tribal population are also at higher risk of facing various public health issues (Kumar et al., 2020). Seven tribal predominated districts Kalahandi, Kandhamal, Nabarangpur, Mayurbhanj, Keonjhar, Sambalpur, and Sundargarh were selected for the study (Figure 1). A population-based, age-stratified, cross-sectional study design was adopted for the study.

### 2.2. Sampling framework

A multi-stage random sampling method was used in each district. Villages within each district (clusters) were selected through probability proportionate to size method. Sample size for each district was calculated to be 395 (rounded off to 400) with an assumption of an expected prevalence of 50% (reported in previous surveys), relative precision of 16%, design effect of 2.5, and non-response rate of 10% for a 95% level of confidence. Ten clusters in each district (total 70 clusters) were selected using the PPS methodology using household population size from the census. From each cluster, at least 40 individuals (4 with age 6–9 years, 8 with age 10–17 years and 28 aged 18 years and above) were enrolled in the survey (Kshatri et al., 2022). Enrolment of a minimum number of individuals in each age group was ensured so that the overall distribution of the sampled population will be comparable to the age structure of the population of the state. Therefore, a minimum of 40 tribal individuals from each cluster and 400 individuals from 10 clusters of each tribal predominated district were enrolled except for three districts Mayurbhanj, Sambalpur, and Sundargarh (due to lack of sufficient volume of samples).

### 2.3. Laboratory investigation

All the sera were subjected to HBsAg (a marker of chronic infection) screening, using commercial enzyme-linked immunosorbent assay HEPALISA (J Mitra & Co. Pvt. Ltd., India) with a sensitivity and specificity of 100 and 99.92%, respectively. Specimens that were equivocal on repeat testing were considered as negative. All the positive HBsAg sera were tested for the presence of HBV DNA (to check the active HBV viral replication) and viral load measurement in the patient using cobas^®^ HBV (Roche Diagnostics India Pvt. Ltd.) with a specificity of 100%. The positive HBsAg sera were also tested for the different biochemical parameters like SGOT, SGPT, ALP, total bilirubin, direct bilirubin, and albumin to check the liver function test of the infected patients using automated biochemistry analyzer EM-360 (ERBA Diagnostics Mannheim GmbH) and reagents provided by Transasia Biochemical Ltd.

### 2.4. Statistical analysis

Descriptive analysis of the findings based on frequency, proportion, HBsAg positivity, and prevalence of Hepatitis B was calculated in accordance with the socio-demographic distribution. Hepatitis B virus DNA obtained from the HBsAg positive participants was log transformed and calculated in mean ± SD (IU/mL). The characteristics of study participants were described as proportions along with 95% CI. Statistical analysis was carried out in IBM Statistical Package for the Social Sciences (SPSS) software version 21.

### 2.5. Ethical approval

All the participants were asked to provide written informed consent before participation in the study. The study was approved by the Institutional Human Ethics Committee.

## 3. Results

A total of 2,737 sera specimens collected from tribal population aged 6 years and above were tested for HBsAg. This included 279 (10.2%) sera from children aged 6–9 years, 450 (16.4%) from participants aged 10–15 years, 1,497 (54.7%) from participants aged 16–49 years, and 511 (18.7%) participants aged 50 years and above. About 1,176 (42.9%) of the sera tested were from male (Table 1). The district-wise and tribe-wise distribution of participants is provided in Tables 1, 2, respectively. Of the 2,737 sera tested, 70 (2.56%; 95%CI: 2.01–3.24) were positive for HBsAg. The PVTGs had a significantly higher prevalence of HBsAg than other STs (Table 1).

**Table 1:** Demographic details of the participants included.

Variables	Frequency	Proportion (%)	HBsAg positive n (%)	Prevalence (95% CI)	HBV DNA (mean ± SD) (IU/mL)
Age					
6–9	279	10.2	5 (1.79)	0.66–4.37	8.33 ± 8.24
10–15	450	16.4	11 (2.44)	1.29–4.46	19.24 ± 21.19
16–49	1,497	54.7	44 (2.94)	2.17–3.96	17.44 ± 18.79
50 and above	511	18.7	10 (1.96)	0.99–3.69	8.09 ± 8.56
Gender					
Male	1,176	43.0	26 (2.21)	1.48–3.27	16.17 ± 17.10
Female	1,561	57.0	44 (2.82)	2.08–3.80	17.74 ± 18.93
Districts					
Kalahandi	514	18.8	24 (4.67)	3.08–6.97	18.17 ± 19.19
Kandhamal	488	17.8	23 (4.71)	3.07–7.09	10.08 ± 10.69
Nabarangpur	486	17.8	3 (0.62)	0.16–1.95	2.08 ± 2.48
Keonjhar	480	17.5	9 (1.88)	0.92–3.66	17.64 ± 18.42
Mayurbhanj	270	9.9	2 (0.74)	0.13–2.94	–
Sambalpur	195	7.1	1 (0.51)	0.03–3.26	–
Sundargarh	304	11.1	8 (2.63)	1.23–5.32	15.96 ± 16.77
Ethnicity					
Scheduled tribes	2,409	88.0	39 (1.62)	1.17–2.23	17.70 ± 18.96
PVTG	328	12.0	31 (9.45)	6.61–13.27	16.70 ± 17.89

**Table 2:** The HBsAg positivity among various tribal community.

Tribes	Districts present	Population covered	HBsAg positive n (%)
Scheduled tribe			
Bhatara	Nabarangpur	22	0
Bhatra	Nabarangpur	340	3 (0.88)
Bhuiya	Keonjhar, Sundergarh, Mayurbhanj, Sambalpur	36	0
Bhuyan	Keonjhar, Sundergarh, Mayurbhanj, Sambalpur	64	2(3.13)
Gond	Kalahandi, Nabarangpur	100	6 (6.00)
Gondo	Keonjhar, Sambalpur, Kalahandi	50	2 (4.00)
Juang	Keonjhar	64	0
Khond	Kandhamal, Kalahandi	333	12 (3.60)
Kisan	Sundergarh, Sambalpur	152	0
Kol	Keonjhar, Mayurbhanj	47	2 (4.26)
Kolha	Mayurbhanj, Keonjhar	47	0
Kond	Kandhamal, Kalahandi	176	1 (0.57)
Kora	Keonjhar	21	0
Munda	Sundergarh, Sambalpur, Keonjhar	128	1 (0.78)
Oraon	Sundergarh, Sambalpur, Keonjhar	65	0
Rajuar	Mayurbhanj	16	1 (6.25)
Santal	Mayurbhanj, Keonjhar	34	0
Saora	Kalahandi	25	0
Savar	Kalahandi	297	7 (2.36)
Sounti_Bhumia	Keonjhar, Mayurbhanj	29	0
Others (Bathudi, Bhumij, Binjhal, Dadua, Didayi, Gadaba, Ghara, Kawar, Kharia, Kharwar, Korua, Koya, Madia, Mundari, Paroja, etc.)	Keonjhar, Sundergarh, Mayurbhanj, Sambalpur, Kalahandi, Kandhamal, Nabarangpur	366	2 (0.55)
PVTG’s			
Dongria_Kondh	Kalahandi	1	1 (100.00)
Kutia_Khond	Kalahandi, Kandhamal	141	20 (14.18)
Lanjia_Saora	Kalahandi	1	0
Paudi_Bhuyan	Sundargarh, Keonjhar	165	10 (6.06)
Saora	Kalahandi	17	0

HBsAg positivity was recorded as 1.79% (*n* = 5); 2.44% (*n* = 16); 2.94% (*n* = 44); and 1.96% (*n* = 10) in the age group of 6–9 years, 10–15 years, 16–49 years, and above 50 years, respectively. The HBsAg positivity was detected as 14.18 and 6.06% among the PVTGs, Kutia Khond, and Paudi Bhuyan tribes. Among the Scheduled tribes, the prevalence of HBsAg was highest among Rajuar (6.25%) followed by Gond (6.0%), Kol (4.26%), Gondo (4%), Khond (3.6%), Bhuyan (3.13%), and Savar (2.36%) (Supplementary Table S1).

Serological evidence of HBV infection in the different age classes and gender is presented in Table 1. Kandhamal district showed the highest positivity (*n* = 23; 4.7%) of HBsAg followed by Kalahandi (*n* = 24; 4.67%), Sundargarh (*n* = 8; 2.63%), and Keonjhar (*n* = 9; 1.88%). The rest of the 3 districts showed a prevalence of less than 1%.

Among the 70 HBsAg positive individuals, 30 (42.9%) were found positive for HBV DNA. The viral load among HBsAg positives ranged between 0.10 × 10^2^–6.84 × 10^8^ IU/mL (Supplementary Table S1). The viral load among the HBsAg positives in the age group of 6–9 years was 0.10 × 10^2^–7.47 × 10^3^ IU/mL. Among the Kutia Khond PVTGs, 8 out of 20 HBsAg positive (40%) showed the presence of HBV DNA with viral load of 0.4 × 10^2^–1.34 × 10^5^ IU/mL. Six out of 10 (60%) HBsAg positive Paudi Bhuyan PVTGs showed the presence of HBV DNA with viral load of 0.17 × 10^2^–4.29 × 10^7^ IU/mL. Both the PVTGs were first time surveyed for HBsAg prevalence and showed high viral load indicating a high potential to transmit the virus.

All the HBsAg positive individual had normal SGPT and 11 individuals had abnormal SGOT (Supplementary Table S1). Among these 11 individuals, 6 had the presence of HBV DNA. Eighteen individuals with HBsAg had abnormal ALP and 8 among them had the presence of HBV DNA. Among all the HBsAg positive individuals, mean SGOT, ALP, Total Bilirubin, and Albumin levels were 64.96 U/L, 255.67 U/L, 0.75 mg/dL, and 5.27 g/dL, respectively. Direct Bilirubin were normal among all the HBsAg positive individuals.

## 4. Discussion

By 2030, the WHO Global Health Sector Strategy aims to eradicate hepatitis through increased prevention, testing, and treatment (WHO Factsheet-b- World Hepatitis Day, 2016).

In this statewide representative serosurvey conducted in 7 districts of Odisha inhabited majorly by scheduled tribe and PVTG population, we detected about 3% of children in the age group 6–15 years were carriers of HBV. The study first time reports the prevalence of HBsAg among the 35 STs and 5 PVTGs residing in Odisha, the eastern state of India. The prevalence of HBsAg is used to classify the endemicity of HBV infection as well as to quantify the burden of disease attributable to HBV infection using mathematical models (Murhekar et al., 2020). The prevalence of HBsAg reported in our serosurvey was lower than reported elsewhere among the tribal population from different parts of the country (Bhattacharya et al., 2015; Sharma et al., 2019; Manjiyil and Konikkara, 2021). Using population-weights, it is estimated that the point-prevalence of hepatitis B among non-tribal populations is 2.4% [95% CI: 2.2–2.7] and among tribal populations it is 15.9% [95% CI: 11.4–20.4%] (Premkumar and Kumar, 2021). The prevalence, however, varies with geographic regions with the highest prevalence in northeastern and northern states (Dwibedi et al., 2014; Sharma et al., 2019). A number of clusters of HBV infections have been detected in regions like Ladakh (12.7%), Arunachal Pradesh (21.2%), and Nicobarese (23.3%), Shompen (37.8%), and Jarawa (65%) tribes of the Andaman and Nicobar Islands (Premkumar and Kumar, 2021). In the present study, the prevalence of HBsAg was found highest in the age group of 16–49 years followed by 10–15 years, which may be attributed to the fact that these individuals might have not got the opportunity to be vaccinated in childhood, or may be due to increased social activities and occupational exposure, indicating that this group of people is at accumulated risk of hepatitis B infection (Sun et al., 2021).

Similar to previous studies from India (Bhattacharya et al., 2015), our findings revealed a higher prevalence of HBsAg among the female population. India introduced HB vaccine in the Universal Immunization during 2011–2012 (phase-II) in seven states of India including Odisha (Lahariya et al., 2013). Over the period of 2008–2016, the hepatitis B vaccine coverage in India has increased from 28.9 to 62.8% (Lahariya et al., 2013; Government of India, 2016). In our study, 1.8% of the participants in the age group of 6–9 years (born after the introduction of HB vaccine) were HBsAg positive, which might be breakthrough infections. Regular monitoring of humoral immunity for HBV and revaccination programs in cases with immunity loss are necessary (Sintusek et al., 2022). In our study, 42.9% of HBsAg positive individuals showed the presence of HBV DNA and the high viral load ranged 0.10 × 10^2^–6.84×10^8^ IU/mL which indicates a high potential to transmit the virus and corroborates an earlier report (Dwibedi et al., 2014).

In the current study, two of these PVTGs, Kutia Khond (Kalahandi & Kandhamal) and Paudi Bhuyan (Sundargarh & Keonjhar), showed a higher prevalence of HBV infection, although all five PVTGs included in the study share similar socio-cultural aspects, geographical location, and relative isolation from the general population. The present study first-time documents the prevalence of HBsAg among the major tribal population residing in the eastern state of the country. To effectively allocate resources in order to prevent, test for, and treat viral hepatitis, these updated data on HBV prevalence will be useful for assessing mortality from HBV associated cirrhosis in state level. Based on the varying prevalence of HBV in certain populations, more effort and resources must be devoted to educating the community and children on Hepatitis B and its serious complications (Hyun et al., 2018).

Our study has three key limitations, firstly, in the main survey, we did not include children younger than 5 years of age for logistical reasons. Secondly, we did not collect information about hepatitis B vaccination from the participants, considering issues regarding parental recall and non-availability of vaccination cards and lastly inability to test different other markers of Hepatitis B infection due to scarcity of sample volume. The sampling technique and sample size calculation were not customized for Hepatitis B seroprevalence survey among the tribal population. To mitigate this, we have provided 95% CI for the estimates. However, since weight were not used for balancing demographic representation, the estimates may not be truly representative.

## 5. Conclusion

The study documents high rates of HBV infection in some of the particularly vulnerable tribal communities residing in Odisha, eastern India. The study findings could be considered as an interim assessment of the status of Hepatitis B infection among the tribal communities and PVTGs residing in Odisha state. About 2% of the children born after the introduction of Hep B vaccine were positive for HBsAg, indicating the need to improve the coverage of three doses of Hepatitis B vaccine in India. The study also highlights the need for a statewide survey of Hepatitis B infection and risk factors, coverage and impact of the Hep B vaccination program introduced in 2010–2011 in Odisha with special reference to the ST and PVTG population of the state.

## Data availability statement

The raw data supporting the conclusions of this article will be made available by the authors, without undue reservation.

## Ethics statement

The studies involving human participants were reviewed and approved by the Institutional Ethics Committee of ICMR-Regional Medical Research Centre, Bhubaneswar. Written informed consent to participate in this study was provided by the participants’ legal guardian/next of kin.

## Author contributions

SP, DB, JK and HB were involved in the concept, planning, and formulation of the study. JK, MP, HC, GD, and NA were involved in data collection and data interpretation. DP, SS, AS, and IM were responsible for laboratory testing. HB and MP performed the data analysis and prepared the initial draft. DB and SP supervised the study. All authors have read and approved the final version of the manuscript.

## Conflict of interest

The authors declare that the research was conducted in the absence of any commercial or financial relationships that could be construed as a potential conflict of interest.

## Publisher’s note

All claims expressed in this article are solely those of the authors and do not necessarily represent those of their affiliated organizations, or those of the publisher, the editors and the reviewers. Any product that may be evaluated in this article, or claim that may be made by its manufacturer, is not guaranteed or endorsed by the publisher.
