# Supplementary material for: Hepatitis B virus infection among the tribal and particularly vulnerable tribal population from an eastern state of India: Findings from the serosurvey in seven tribal dominated districts, 2021–2022
Source: Front Microbiol. 2023 Mar 6;14:1039696. doi: 10.3389/fmicb.2023.1039696 (PMC10025534; doi:10.3389/fmicb.2023.1039696)
Supplement: Supplementary file 1 [file Table_1.docx]

**Table S1. Viral load and biochemical parameters among the HBsAg positive individuals**

| **Sample No.** | **Age**  **(in years)** | **Gender** | **Ethnicity** | **District** | **HBV**  **Viral Load**  **(IU/ml)** | **SGPT Normal Range 0.0-45.0 U/L** | **SGOT Normal Range 0.0 -35.0 U/L** | **ALP Normal Range 53-128 U/L** | **BID Normal Range 0.0-0.20 mg/dl** | **BIT Normal Range 0.0- 0.20 mg/dl** | **ALBUMIN Normal Range 3.50-5.20 g/dl** |
| --- | --- | --- | --- | --- | --- | --- | --- | --- | --- | --- | --- |
| 1 | 6 | Male | Ghara | Mayurbhanj | - | 1.2 | 12.3 | 104 | 0.01 | 0.08 | 4.9 |
| 2 | 36 | Male | Bhumij | Mayurbhanj | - | 18.9 | 26.3 | 61 | 0.03 | 0.06 | 5.15 |
| 3 | 35 | Female | Gondo | Keonjhar | - | 3.1 | 50 | 102 | 0.02 | 0.16 | 4.75 |
| 4 | 35 | Female | Rajuar | Keonjhar | 7.41 x 10^2^ | 43.1 | 12.7 | 249 | 0.03 | 0.05 | 4.69 |
| 5 | 19 | Female | Kol | Keonjhar | - | 13 | 23.4 | 93 | 0.03 | 0.1 | 4.23 |
| 6 | 19 | Male | Kol | Keonjhar | 2.68 x 10^6^ | 4.7 | 12 | 205 | 0.02 | 0.18 | 4.85 |
| 7 | 22 | Male | Paudi_Bhuyan | Keonjhar | - | 8.1 | 51 | 75 | 0.04 | 0.07 | 5.07 |
| 8 | 46 | Female | Paudi_Bhuyan | Keonjhar | 4.28 x 10^2^ | 7.1 | 12 | 137 | 0.17 | 0.07 | 5.14 |
| 9 | 15 | Female | Paudi_Bhuyan | Keonjhar | 2.26 x 10^8^ | 11.5 | 18.5 | 322 | 0.02 | 0.07 | 4.55 |
| 10 | 14 | Female | Paudi_Bhuyan | Keonjhar | - | 12.7 | 18.8 | 82 | 0.03 | 0.08 | 4.89 |
| 11 | 9 | Female | Paudi_Bhuyan | Keonjhar | 0.17 x 10^2^ | 30.1 | 26.6 | 127 | 0.02 | 0.08 | 4.52 |
| 12 | 12 | Female | Kutia_Khond | Kalahandi | - | 9.3 | 19.5 | 90 | 0.02 | 0.06 | 4.3 |
| 13 | 9 | Male | Kutia_Khond | Kalahandi | 4.97 x 10^3^ | 8.1 | 25.7 | 121 | 0.05 | 0.05 | 4.84 |
| 14 | 9 | Female | Kutia_Khond | Kalahandi | - | 15.5 | 9.1 | 90 | 0.03 | 0.05 | 4.25 |
| 15 | 46 | Female | Kond | Kalahandi | - | 7.1 | 29.2 | 77 | 0.03 | 0.05 | 4.49 |
| 16 | 12 | Female | Kutia_Khond | Kalahandi | - | 11.2 | 24 | 80 | 0.03 | 0.05 | 4.55 |
| 17 | 15 | Female | Dongria_Khond | Kalahandi | - | 2.3 | 19.5 | 69 | 0.19 | 0.04 | 4.29 |
| 18 | 26 | Female | Gond, | Kalahandi | - | 6.2 | 27.3 | 79 | 0.02 | 0.05 | 4.78 |
| 19 | 18 | Female | Gond, | Kalahandi | 6.84 x 10^8^ | 3.8 | 74.1 | 219 | 0.05 | 0.11 | 4.36 |
| 20 | 27 | Female | Gond, | Kalahandi | - | 7.1 | 24.4 | 72 | 0.03 | 0.04 | 4.34 |
| 21 | 66 | Male | Gond, | Kalahandi | - | 3.4 | 161 | 84 | 0.04 | 0.05 | 4.01 |
| 22 | 62 | Female | Gond, | Kalahandi | 1.77 x 10^3^ | 2 | 40.6 | 69 | 0.15 | 0.06 | 4.05 |
| 23 | 22 | Female | Gond, | Kalahandi | - | 8.1 | 13.6 | 58 | 0.05 | 0.06 | 3.73 |
| 24 | 34 | Female | Bhatra | Nabarangpur | 0.17 x 10^2^ | 10.5 | 68.9 | 103 | 0.04 | 0.08 | 4.67 |
| 25 | 60 | Male | Savar | Kalahandi | - | 3.7 | 22.1 | 101 | 0.02 | 0.06 | 3.75 |
| 26 | 80 | Female | Savar | Kalahandi | 0.14 x 10^2^ | 6.1 | 22.7 | 78 | 0.02 | 0.11 | 4.29 |
| 27 | 32 | Male | Khond | Kalahandi | - | 2.5 | 44.5 | 66 | 0.04 | 0.11 | 4.49 |
| 28 | 33 | Male | Khond | Kalahandi | 9.07 x 10^7^ | 16.4 | 35.7 | 200 | 0.06 | 0.06 | 4.65 |
| 29 | 37 | Male | Khond | Kalahandi | 0.3x 10^2^ | 13 | 14 | 129 | 0.05 | 0.06 | 4..04 |
| 30 | 29 | Male | Khond | Kalahandi | 9.24 x 10^2^ | 5.9 | 11.4 | 239 | 0.06 | 0.09 | 4.43 |
| 31 | 46 | Female | Bhatra | Nabarangpur | - | 3.1 | 15.3 | 74 | 0.03 | 0.04 | 4.56 |
| 32 | 52 | Male | Savar | Kalahandi | 1.10 x 10^4^ | 5.1 | 24.4 | 122 | 0.08 | 0.06 | 3.98 |
| 33 | 13 | Male | Savar | Kalahandi | - | 33.1 | 27.3 | 75 | 0.03 | 0.04 | 4.43 |
| 34 | 36 | Female | Savar | Kalahandi | - | 1.9 | 13.6 | 68 | 0.06 | 0.08 | 4.89 |
| 35 | 35 | Female | Savar | Kalahandi | 3.53 x 10^6^ | 0.3 | 99.1 | 84 | 0.02 | 0.04 | 4.14 |
| 36 | 11 | Male | Khond | Kalahandi | - | 21.7 | 4.9 | 82 | 0.06 | 0.05 | 4.86 |
| 37 | 55 | Female | Savar | Kalahandi | - | 5.3 | 19.2 | 85 | 0.08 | 0.05 | 4.85 |
| 38 | 37 | Female | Bhatra | Nabarangpur | - | 5.6 | 9.4 | 102 | 0.01 | 0.04 | 4.69 |
| 39 | 31 | Male | Kutia_Khond | Kandhamal | 2.04 x 10^3^ | 5.9 | 29.6 | 327 | 0.04 | 0.05 | 5.02 |
| 40 | 26 | Male | Kutia_Khond | Kandhamal | 1.34 x 10^5^ | 12.4 | 23.1 | 127 | 0.11 | 0.15 | 4.51 |
| 41 | 22 | Female | Kutia_Khond | Kandhamal | 0.5 x 10^2^ | 9 | 13.6 | 229 | 0.04 | 0.08 | 4.69 |
| 42 | 50 | Female | Kutia_Khond | Kandhamal | 3.28 x 10^2^ | 13.3 | 7.8 | 404 | 0.04 | 0.05 | 4.24 |
| 43 | 50 | Female | Kutia_Khond | Kandhamal | - | 8.1 | 24.7 | 78 | 0.05 | 0.07 | 3.57 |
| 44 | 30 | Female | Kutia_Khond | Kandhamal | 0.4 x 10^2^ | 2.8 | 22.7 | 108 | 0.07 | 0.12 | 3.84 |
| 45 | 19 | Female | Kutia_Khond | Kandhamal | - | 8.7 | 16.9 | 78 | 0.02 | 0.07 | 4.42 |
| 46 | 11 | Female | Kutia_Khond | Kandhamal | - | 6.8 | 11.7 | 80 | 0.19 | 0.16 | 3.99 |
| 47 | 9 | Female | Kutia_Khond | Kandhamal | 7.47 x 10^3^ | 3.7 | 37.7 | 163 | 0.03 | 0.06 | 3.97 |
| 48 | 35 | Female | Kutia_Khond | Kandhamal | - | 5.3 | 14 | 76 | 0.03 | 0.07 | 4.15 |
| 49 | 26 | Female | Kutia_Khond | Kandhamal | - | 9.6 | 17.9 | 114 | 0.05 | 0.13 | 4.28 |
| 50 | 50 | Male | Kutia_Khond | Kandhamal | - | 3.7 | 20.1 | 103 | 0.04 | 0.04 | 3.93 |
| 51 | 33 | Male | Kutia_Khond | Kandhamal | - | 11.5 | 18.5 | 105 | 0.03 | 0.13 | 4.01 |
| 52 | 44 | Female | Kutia_Khond | Kandhamal | 4.21 x 10^4^ | 2.3 | 34.4 | 295 | 0.01 | 0.08 | 4.29 |
| 53 | 11 | Male | Kutia_Khond | Kandhamal | - | 3.7 | 17.2 | 73 | 0.04 | 0.07 | 4.13 |
| 54 | 14 | Female | Kutia_Khond | Kandhamal | - | 4 | 15.9 | 112 | 0.01 | 0.74 | 4.5 |
| 55 | 30 | Female | Khond | Kandhamal | - | 0.2 | 19.5 | 102 | 0.04 | 0.69 | 4.04 |
| 56 | 18 | Male | Khond | Kandhamal | - | 20.5 | 23.4 | 69 | 0.04 | 0.07 | 4.17 |
| 57 | 26 | Female | Khond | Kandhamal | 0.86 x 10^2^ | 1.8 | 22.4 | 125 | 0.04 | 0.07 | 3.98 |
| 58 | 30 | Female | Khond | Kandhamal | - | 3.1 | 12.7 | 88 | 0.09 | 0.07 | 4.01 |
| 59 | 27 | Female | Khond | Kandhamal | - | 6.8 | 15.9 | 105 | 0.02 | 0.08 | 4.73 |
| 60 | 50 | Male | Khond | Kandhamal | - | 9.9 | 16.9 | 122 | 0.03 | 0.08 | 4.29 |
| 61 | 37 | Male | Khond | Kandhamal | 2.86 x 10^4^ | 4.3 | 10.7 | 172 | 0.05 | 0.06 | 4.37 |
| 62 | 44 | Male | Gondo | Sambalpur | 2.41 x 10^2^ | 12.1 | 7.1 | 266 | 0.08 | 0.05 | 4.21 |
| 63 | 25 | Female | Munda | Sundargarh | 0.10 x 10^2^ | 4.7 | 10.7 | 259 | 0.04 | 0.07 | 4.31 |
| 64 | 41 | Female | Bhuyan | Sundargarh | - | 9.3 | 13 | 105 | 0.09 | 0.07 | 4.24 |
| 65 | 38 | Female | Bhuyan | Sundargarh | 0.15 x 10^2^ | 0.9 | 22.4 | 464 | 0.04 | 0.07 | 4.05 |
| 66 | 28 | Male | Paudi_Bhuyan | Sundargarh | 1.96 x 10^3^ | 13 | 19.2 | 71 | 0.02 | 0.08 | 4.23 |
| 67 | 26 | Female | Paudi_Bhuyan | Sundargarh | - | 9.9 | 24.4 | 83 | 0.04 | 0.08 | 4.37 |
| 68 | 11 | Female | Paudi_Bhuyan | Sundargarh | - | 6.2 | 9.7 | 91 | 0.06 | 0.06 | 5.27 |
| 69 | 32 | Male | Paudi_Bhuyan | Sundargarh | 5.10 x 10^3^ | 10.9 | 6.5 | 109 | 0.05 | 0.05 | 4.01 |
| 70 | 40 | Male | Paudi_Bhuyan | Sundargarh | 4.29 x 10^7^ | 1.2 | 52 | 323 | 0.01 | 0.08 | 4.9 |
